# Circulating Exhausted PD-1^+^CD39^+^ Helper CD4 T Cells Are Tumor-Antigen-Specific and Predict Response to PD-1/PD-L1 Axis Blockade

**DOI:** 10.3390/cancers14153679

**Published:** 2022-07-28

**Authors:** Carlos Martinez-Gomez, Marie Michelas, Clara-Maria Scarlata, Anna Salvioni, Carlos Gomez-Roca, Victor Sarradin, Françoise Lauzéral-Vizcaino, Virginie Féliu, Agnès Dupret-Bories, Gwénaël Ferron, Jérôme Sarini, Christel Devaud, Jean-Pierre Delord, Camille-Charlotte Balança, Alejandra Martinez, Maha Ayyoub

**Affiliations:** 1 Centre de Recherches en Cancérologie de Toulouse, Inserm, CNRS, Université Toulouse III-Paul Sabatier, Université de Toulouse, 31037 Toulouse, France; c-martinezgomez@o-lambret.fr (C.M.-G.); marie.michelas@inserm.fr (M.M.); clara-maria.scarlata@inserm.fr (C.-M.S.); anna.salvioni@inserm.fr (A.S.); gomez-roca.carlos@iuct-oncopole.fr (C.G.-R.); victor.sarradin@inserm.fr (V.S.); francoise.lauzeral-vizcaino@inserm.fr (F.L.-V.); virginie.feliu@inserm.fr (V.F.); christel.devaud@inserm.fr (C.D.); delord.jean-pierre@iuct-oncopole.fr (J.-P.D.); balanca.camillecharlotte@gene.com (C.-C.B.); martinez.alejandra@iuct-oncopole.fr (A.M.); 2 Department of Surgery, IUCT-Oncopole, 31059 Toulouse, France; dupret-bories.agnes@iuct-oncopole.fr (A.D.-B.); ferron.gwenael@iuct-oncopole.fr (G.F.); sarini.jerome@iuct-oncopole.fr (J.S.); 3 Immune Monitoring Core Facility, IUCT-Oncopole, 31059 Toulouse, France; 4 Department of Medical Oncology, IUCT-Oncopole, 31059 Toulouse, France

**Keywords:** CD4 T-cell response, tumor antigens, cancer immunotherapy, circulating biomarkers

## Abstract

**Simple Summary:**

Not all cancer patients receiving immunotherapy by immune checkpoint blockade experience a clinical benefit. Our study was aimed at identifying biomarkers that could guide the selection of immunotherapy-responsive patients. Immunotherapy targets two major populations of lymphocytes: CD8 T cells, which directly kill tumor cells, and CD4 T cells, which provide help to CD8 T cells, the role of which in clinical responsiveness to immunotherapy has been less explored. We identified, in the blood of cancer patients, a population of CD4 T cells expressing inhibitory receptors targeted by immunotherapy. We showed that these cells were activated and proliferating, indicating their potential involvement in ongoing immune responses. Accordingly, we showed that they were specific for tumor antigens. In a prospective cohort, we showed that high proportions of these cells prior to therapy were associated with a response to immunotherapy.

**Abstract:**

Tumor-infiltrating exhausted PD-1^hi^CD39^+^ tumor-antigen (Ag)-specific CD4 T cells contribute to the response to immune checkpoint blockade (ICB), but their circulating counterparts, which could represent accessible biomarkers, have not been assessed. Here, we analyzed circulating PD-1^+^CD39^+^ CD4 T cells and show that this population was present at higher proportions in cancer patients than in healthy individuals and was enriched in activated HLA-DR^+^ and ICOS^+^ and proliferating KI67^+^ cells, indicative of their involvement in ongoing immune responses. Among memory CD4 T cells, this population contained the lowest proportions of cells producing effector cytokines, suggesting they were exhausted. In patients with HPV-induced malignancies, the PD-1^+^CD39^+^ population contained high proportions of HPV Ag-specific T cells. In patients treated by ICB for HPV-induced tumors, the proportion of circulating PD-1^+^CD39^+^ CD4 T cells was predictive of the clinical response. Our results identify CD39 expression as a surrogate marker of circulating helper tumor-Ag-specific CD4 T cells.

## 1. Introduction

Immunotherapy by immune checkpoint blockade (ICB) has transformed cancer care; yet, even in ICB-sensitive malignancies, not all patients experience a clinical benefit and identification of predictive biomarkers is awaited [1]. Their identification is tightly dependent upon the understanding of the cellular and molecular mechanisms directly or indirectly targeted by ICB and underlying their effectiveness [2]. Biomarkers can be appraised at the tumor site or in circulation. In situ cell populations are involved in immune processes directly related to tumor development and progression, but are not systematically accessible. In contrast, circulating immune cells are readily reachable through less invasive interventions [3]. However, a major challenge is the identification, among circulating immune cells, of those impacted by the tumor and manifesting the ongoing anti-tumor immune response.

Exhausted tumor-antigen (Ag)-specific CD8 T cells at the tumor site were identified by us and others as the major target of therapeutic PD-1/PD-L1 axis blocking monoclonal antibodies [4,5]. We showed that anti-PD-1 had a dual effect on one hand on tumor-infiltrating terminally exhausted CD8 T cells, which recover their effector functions, and on the other, on circulating early exhausted tumor-specific CD8 T cells, which proliferate to replenish the tumor site [4]. These results and those from other groups highlighted the importance of both circulating and tumor-infiltrating CD8 T cells in the response to ICB [4,6]. In support of the importance of peripheral adaptive immunity, early proliferation of circulating PD-1^+^ CD8 T cells was shown to be associated with anti-PD-1 efficacy in lung cancer and proposed as a biomarker of response [7]. These studies highlight the importance of both the tumoral and circulating compartments in understanding patient responsiveness to ICB.

In addition to CD8 T cells, which directly recognize and kill tumor cells, we found that terminally exhausted PD-1^hi^CD39^+^ CD4 T cells at the tumor site are tumor-Ag-specific and lead to DC maturation and to Ag-specific CD8 T-cell proliferation following PD-1 blockade [8]. The tumor Ag specificity of CD39^+^ tumor-infiltrating CD4 T cells was also demonstrated in human-papillomavirus (HPV)-induced malignancies [9]. Later studies further underlined the role of exhausted tumor-infiltrating tumor-Ag-specific CD4 T cells in the effector phase of the anti-tumor immune response [10,11,12,13,14].

Given the importance of CD4 T-cell help for CD8 T-cell proliferation, both at the tumor site and in the periphery [8,15,16], here, we investigated the expression of PD-1 and CD39 in circulating CD4 T cells in cancer patients bearing HPV-induced malignancies. We present data showing that PD-1^+^CD39^+^ CD4 T cells in circulation reflect an ongoing adaptive immune response, which, in cancer patients with HPV-induced tumors, was directed against HPV Ags and was predictive of the response to PD-1/PD-L1 axis blockade.

## 2. Materials and Methods

### 2.1. Patient and Healthy Donor Samples

Peripheral blood and tumor samples were collected from patients with head and neck squamous cell carcinoma (HNSCC) or cervical cancer (CC) at the time of surgery for primary disease or for recurrence at the Institut Universitaire du Cancer de Toulouse—Oncopole (IUCT-O) in accordance with the Declaration of Helsinki, upon approval by the Institutional Review Board (DECIdE protocol, NCT03958240) and signed informed consent. The study included 34 patients with localized CC with squamous and adenocarcinoma histology, of which 33 were HPV 16 and/or 18 positive, and 29 patients with locoregional newly diagnosed or recurrent HNSCC, among them 19 had HPV positive tumors. All patients had histologically documented tumors, were ≥18 years old at the time of study entry, were followed within a standard of care procedure, and had an ECOG performance status of 0–2. The exclusion criteria were as follows: known history of another primary malignancy in the previous 5 years; known history of a positive test for hepatitis B, hepatitis C, human immunodeficiency, or Hanta viruses; any condition contraindicated with blood sampling procedures; pregnancy or breast-feeding; active suspected or prior documented autoimmune disease; or the use of immunosuppressive medication. Patients did not receive any therapy during the three months prior to study entry. Pre- and posttreatment blood samples were obtained from a second cohort of 13 patients with HPV-induced cancers (CC, *n* = 7; anal cancer, *n* = 6) receiving ICB therapy with PD-1/PD-L1–blocking agents (anti-PD-1, *n* = 7; anti-PD-L1, *n* = 6) to treat metastatic disease at IUCT-O in accordance with the Declaration of Helsinki, upon approval by the Institutional Review Board (MINER protocol, NCT03514368) and signed informed consent. Pre- and posttreatment blood samples were obtained prior to the first (T0) and third (T1) ICB dosing, respectively. Response to therapy was evaluated by iRECIST criteria. Progressive disease (PD) was defined as an increase of >20% of target lesions or appearance and an increase in the size of new lesions in at least two CT scan evaluations performed at least 4 weeks apart. Partial response (PR) was defined as a decrease of >30% in target lesions and complete response (CR) as the disappearance of target and non-target lesions, both in at least two CT scans performed at least 4 weeks apart. Any response other than PD or PR/CR was considered as stable disease (SD). HPV status was determined in HNSCC patients by assessment of the overexpression of p16 in tumor biopsies by immunohistochemistry. PCR-based high-risk HPV type testing was employed for patients diagnosed with CC or anal cancer to identify HPV genotypes. Blood samples from 28 healthy donors (HDs) were obtained from the Etablissement Français du Sang (Toulouse, France).

Peripheral blood mononuclear cells (PBMCs) from patients or HDs were isolated by density gradient sedimentation using Ficoll–Hypaque (Sigma-Aldrich, St. Louis, MO, USA). Tumor samples were rinsed with phosphate-buffered saline (PBS, Sigma-Aldrich); subsequently minced to small pieces, between 2 and 4 mm; dissociated using C-tubes (Miltenyi Biotec, Bergisch Gladbach, Germany) and gentleMACS^TM^ Octo Dissociator (Program MultiC01_01; Miltenyi Biotec) in Iscove’s modified Dulbecco’s medium (IMDM, Sigma-Aldrich); and filtered using a 40 μm nylon mesh (BD Biosciences, Franklin Lakes, NJ, USA). PBMCs and tumor single-cell suspensions were cryopreserved in fetal bovine serum (FBS, Gibco^TM^, Thermo Fisher Scientific, Waltham, MA, USA) containing 10% dimethyl sulfoxide (DMSO, Sigma-Aldrich).

### 2.2. CD4 T Cells’ Purification and Phenotyping

CD4^+^ cells were enriched from PBMCs or tumor single-cell suspensions by positive magnetic selection (CD4 MicroBeads, human, Miltenyi Biotec) using OctoMACS™ Separator and MS Columns (Miltenyi Biotec). Sorted cells were assessed phenotypically by staining with monoclonal antibodies (mAbs) specific for CD3, CD4, CD45RA, CCR7, PD-1, TIGIT, TIM-3, CD28, CD69, CD39, ICOS, and HLA-DR, as indicated, in phosphate-buffered saline (PBS) containing 5% FBS, for 15 min at 4 °C. For intracellular and intra-nuclear staining, cells were fixed and permeabilized with fixation/permeabilization buffer over 45 min at 4 °C, and stained in permeabilization buffer for 45 min at 4 °C with mAbs specific for CTLA-4, KI67, and FOXP3 using the Transcription Factor Staining Buffer Set (eBioscience). mAbs are listed in Supplementary Table S1. Cells were analyzed using BD LSRFortessa™ X20 (BD Biosciences) and data were analyzed using Flowlogic™ software (Miltenyi Biotec).

### 2.3. Sorting and Functional Assessment of CD4 T-Cell Subpopulations

CD4^+^ cells were magnetically sorted from PBMCs as described above. The CD4 negative fraction was used to magnetically sort CD8^+^ cells (CD8 MicroBeads, human, Miltenyi Biotec; OctoMACS™ Separator and MS Columns, Miltenyi Biotec). Sorted CD8 T cells were cryopreserved and the CD4^–^CD8^–^ fraction was used as source of Ag-presenting cells (see below). Sorted CD4 T cells were stained with mAbs specific for CD3, CD4, CDR45RA, PD-1, CD39, CD127, and CD25 (Supplementary Table S1), and memory Tconv (CD3^+^CD4^+^CD8^-^CD45RA^-^CD25^-^CD127^+^) were sorted into PD-1^-^CD39^-^, PD-1^+^CD39^-^, and CD39^+^ subpopulations by flow cytometry (BD FACSAria™ Fusion, BD Biosciences).

For the assessment of HPV Ag-specific CD4 T cells, ex vivo sorted PD-1^-^CD39^-^, PD-1^+^CD39^-^, and CD39^+^ cells were stimulated in vitro with a pool of 37 and 22 overlapping 15 amino acid long peptides encompassing full-length HPV 16 E6 and E7 proteins, respectively, and/or 37 and 24 overlapping 15 amino acid long peptides encompassing full-length HPV 18 E6 and E7 proteins, respectively, according to the HPV type detected in each patient’s tumor (1 µM of each peptide in the pool; Peptide 2.0 Inc, Supplementary Table S2). Stimulation was performed in the presence of autologous irradiated (35 Gy) CD4^-^CD8^-^ PBMCs and recombinant human (rh) IL-2 (50 IU/mL; Miltenyi Biotec) in IMDM supplemented with 1% penicillin-streptomycin solution (Sigma-Aldrich), 1% MEM non-essential amino acids solution (Invitrogen), L-glutamine (2 mM, Invitrogen, Thermo Fisher Scientific, Waltham, MA, USA), and 10% human serum (Institut de Biotechnologies Jacques Boy, Reims, France). Day 20 cultures were stimulated or not with the same HPV peptide pools for 4 h. Brefeldin A (BFA; 3 µg/mL; eBioscience, Thermo Fisher Scientific, Waltham, MA, USA) was added one hour after the beginning of stimulation. Following surface staining with mAbs specific for CD3 and CD4, intracellular staining was performed using mAbs specific for IFN-γ and TNF-α (Supplementary Table S1) in the presence of saponin (Sigma-Aldrich; 0.1% in PBS containing 5% FBS) and cells were analyzed by flow cytometry.

To assess the effector functions of ex vivo sorted PD-1^-^CD39^-^, PD-1^+^CD39^-^, and CD39^+^ cells, they were stimulated in vitro with phorbol 12-myristate 13-acetate (PMA, 100 ng/mL; Sigma-Aldrich) and ionomycin (1 μg/mL; Sigma-Aldrich) or with plate-bound anti-CD3 (1 μg/mL; eBioscience) and anti-CD28 (5 μg/mL; eBioscience) mAbs for 6 h in IMDM, supplemented as detailed above. BFA was added 1 h after the beginning of stimulation and intracellular cytokine staining and flow cytometry analyses were performed as described above.

### 2.4. Acute and Chronic In Vitro Stimulation

CD4^+^ cells were magnetically sorted from PBMCs, as described above, stained with mAbs specific for CD3, CD4, CD45RA, and CCR7 (Supplementary Table S1), and naïve CD4 T cells (CD3^+^CD4^+^CD45RA^+^CCR7^+^) were sorted by flow cytometry (BD FACSMelody™ Cell Sorter, BD Biosciences). An aliquot of sorted naïve cells was stained with anti-CD39 mAbs (Supplementary Table S1) and analyzed by flow cytometry. Sorted naïve cells were stimulated in vitro using plate-bound anti-CD3 and anti-CD28 mAbs, as detailed above, in the presence of rhIL-2 (50 IU/mL) in IMDM supplemented as described above. Twenty-four hours following the beginning of the stimulation, half of the cells were transferred to new plates with no mAbs. Cell cultures in both types of wells, i.e., with or without plate-bound mAbs, were maintained for 10 days in the presence of rhIL-2. Cells maintained in mAb-coated plates were considered ‘chronically stimulated’ and those transferred to uncoated plates ‘acutely stimulated’. At days 2, 4, 7, and 10, aliquots of cells under chronic or acute stimulation conditions were stained using mAbs specific for CD3, CD4, CD45RA, CCR7, PD-1, and CD39 (Supplementary Table S1) and analyzed by flow cytometry.

### 2.5. Statistical Analyses

Normality was assessed using the Shapiro–Wilk test. For normally distributed values, a *t*-test was used for paired or unpaired data. When the values were not normally distributed, the comparison of variables was performed with Wilcoxon or Mann–Whitney tests for paired and unpaired data, respectively. Lines and error bars in scatter plots represent the mean ± SD. The results of statistical analyses are annotated as follows in figures: ns, not significant (*p* ≥ 0.05); *, *p* < 0.05; **, *p* < 0.01; ***, *p* < 0.001; and ****, *p* < 0.0001. All analyses were performed with GraphPad Prism 7 software.

## 3. Results

### 3.1. Circulating CD4 T Cells Contain a PD-1^+^CD39^+^ Population Found at Increased Proportions in Cancer Patients in Comparison with Healthy Individuals

**Figure 1:**
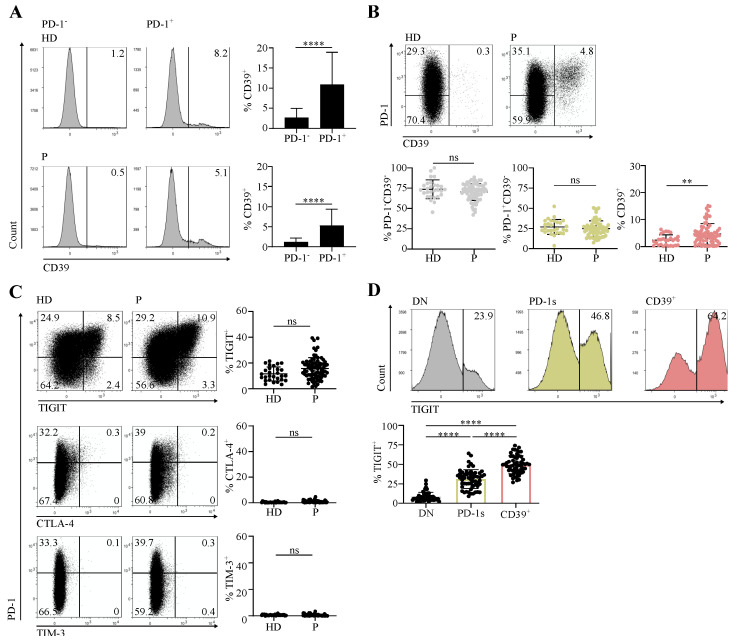
Circulating memory PD-1^+^CD39^+^ CD4 T cells are found at increased proportions in cancer patients in comparison with healthy individuals. CD4 T cells sorted ex vivo from peripheral blood mononuclear cells (PBMCs) of healthy donors (HDs) and cervical cancer (CC) or head and neck squamous cell carcinoma (HNSCC) patients (P) at diagnosis were stained and analyzed by flow cytometry. Data are shown following gating on memory (CD45RA^-^) conventional (FOXP3^-^) CD3^+^CD4^+^ cells (memory Tconv, Supplementary Figure S1). (**A**) Examples of histograms showing CD39 expression in PD-1^-^ and PD-1^+^ memory Tconv populations and summary of the proportions of CD39^+^ cells among the two memory Tconv populations (HD, *n* = 28; P, *n* = 72). (**B**) Dot plots showing PD-1 and CD39 expression and summary of the proportions of the PD-1^-^CD39^-^, PD-1^+^CD39^-^, and CD39^+^ populations among memory Tconv (HD, *n* = 28; P, *n* = 72). (**C**) Dot plots showing expression of TIGIT, CTLA-4, and TIM-3 versus that of PD-1 and summary of the proportions of TIGIT^+^ (HD, *n* = 28; P, *n* = 72), CTLA-4^+^, and TIM-3^+^ (HD, *n* = 28; P, *n* = 63) cells among memory Tconv. (**D**) Histograms showing expression of TIGIT in the PD-1^-^CD39^-^ (DN, double negative), PD-1^+^CD39^-^ (PD-1s, PD-1 single), and CD39^+^ memory Tconv populations and summary of the proportions of TIGIT^+^ cells in the three populations from patients (*n* = 57).

The immunosuppressive ectonucleotidase CD39 participates in extracellular ATP consumption and, in tandem with CD73, in the generation of adenosine [17]. In cancer patients, CD39 is expressed at the tumor site by terminally exhausted tumor-Ag-specific CD8 T cells [4,18,19]. We defined expression of high levels of PD-1 and that of CD39 in tumor-infiltrating CD4 T cells as markers of terminal exhaustion and of tumor Ag specificity [8]. In HPV-induced tumors expressing the highly immunogenic E6 and E7 viral Ags [20], CD39 expression identified HPV Ag-specific CD8 and CD4 tumor-infiltrating lymphocytes (TILs) [9]. Here, we assessed the expression of PD-1 and CD39 in circulating memory (CD45RA^-^) conventional (Tconv; FOXP3^-^) CD4 T cells (Supplementary Figure S1) from head and neck squamous cell carcinoma (HNSCC) and cervical cancer (CC) patients and from healthy donors (HDs). In patients and HDs, CD39 expression was virtually restricted to the PD-1^+^ fraction (Figure 1A). Accordingly, analysis of CD39 versus PD-1 expression identified three major cell subpopulations: PD-1^-^CD39^-^ and PD-1^+^CD39^-^, which we called double negative (DN) and PD-1 single (PD-1s), respectively, as well as CD39^+^ (Figure 1B). Of the three subpopulations, only CD39^+^ cells were found at higher proportions in patients in comparison with HDs (Figure 1B).

We and others have shown that CD4 and CD8 TILs, positive for PD-1, express other immune checkpoints (ICs), including TIGIT, CTLA-4, and TIM-3, and are functionally exhausted [4,5,8,13]. We thus assessed the expression of these ICs in circulating memory Tconv from HDs and cancer patients. We did not detect a significant expression of CTLA-4 and TIM-3 (Figure 1C). In contrast, TIGIT was expressed and the proportions of TIGIT^+^ cells were not different between patients and HDs (Figure 1C). In patients, the proportions of TIGIT^+^ cells were higher in the CD39^+^ subpopulation than among DN and PD-1s cells (Figure 1D).

Our results identify a population of circulating CD39^+^ memory conventional CD4 T cells expressing PD-1 and, for the most part, TIGIT and was present at higher proportions in cancer patients than in healthy individuals.

### 3.2. The Circulating PD-1+CD39+ CD4 T-Cell Subpopulation Is Enriched in Activated and Proliferating Cells That Were at an Early Differentiation Stage

**Figure 2:**
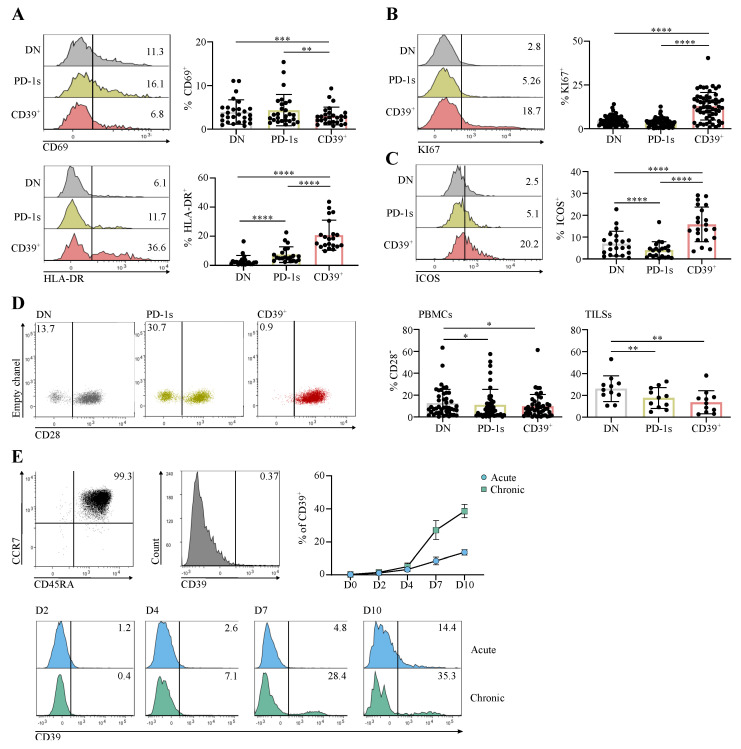
The circulating PD-1+CD39+ CD4 T-Cell subpopulation is enriched in activated and proliferating cells that were at an early differentiation stage. (**A**–**D**) CD4 T cells sorted ex vivo from PBMCs or from tumor cell suspensions (TILs) of CC or HNSCC patients at diagnosis were stained and analyzed by flow cytometry. Expression of the indicated markers is shown in the PD-1^-^CD39^-^ (DN), PD-1^+^CD39^-^ (PD-1s), and CD39^+^ populations gated in memory Tconv. Histograms show the expression of CD69 and HLA-DR (**A**), KI67 (**B**), and ICOS (**C**) in the indicated memory Tconv populations and data obtained for all patients are summarized (CD69 (**A**) *n* = 27; HLA-DR (**A**) *n* = 21; KI67 (**B**) *n* = 56; ICOS (**C**) *n* = 21). Dot plots (**D**) show the expression of CD28 in the indicated memory Tconv populations from PBMCs and the proportions of CD28^-^ cells in each memory Tconv population are summarized (**D**) for PBMCs (*n* = 45) and TILs (*n* = 11). (**E**) Naïve CD4 T cells (CD3^+^CD4^+^CD45RA^+^CCR7^+^), sorted by flow cytometry from healthy donor PBMCs, were stimulated in vitro with anti-CD3 and anti-CD28 mAbs in acute or chronic conditions. CD39 expression was assessed in cultures on days 2, 4, 7, and 10 by flow cytometry following staining with specific mAbs. The dot plot and the histogram in the upper panel show the purity of the sorted naïve population and the absence of CD39 expression, respectively. Histograms in the lower panel show the CD39 expression in D2 to D10 acute and chronic stimulation cultures from 1 HD. Data obtained for all HDs (*n* = 3) are shown as the mean ± SD of CD39 expression at all timepoints for the two stimulation conditions.

The enrichment of PD-1^+^CD39^+^ CD4 T cells in cancer patients was evocative of their involvement in an ongoing immune response. We thus assessed the expression of the early and late T-cell activation markers CD69 and HLA-DR [21], respectively, as well as that of the proliferation marker KI67 according to PD-1 and CD39 expression in memory Tconv from cancer patients. The proportion of CD69^+^ cells was lower in the CD39^+^ subpopulation in comparison with the DN and PD-1s subpopulations (Figure 2A). In contrast, the highest proportions of HLA-DR^+^ and KI67^+^ cells were found within the CD39^+^ subpopulation (Figure 2A,B). The costimulatory molecule ICOS, the expression of which is induced upon T-cell receptor (TCR) and CD28 engagement [22], was also found at higher proportions within the CD39^+^ subpopulation (Figure 2C). These results suggest that the CD39^+^ subpopulation harbors cells that express activation markers and are proliferating, following Ag encounter. In addition, among the three memory Tconv subpopulations, the CD39^+^ subpopulation contained the lowest proportions of CD28^-^ cells (Figure 2D), suggesting that these activated and proliferating cells were at an early differentiation stage. Of note, CD39^+^ memory Tconv among TILs from HNSCC and CC patients also contained lower proportions of CD28^-^ cells in comparison with DN and PD-1s memory Tconv TILs (Figure 2D).

Expression of CD39 in CD8 T cells was shown to result from chronic T-cell stimulation [19]. To investigate if a similar mechanism was involved in CD39 expression in CD4 T cells, we isolated naïve CD4 T cells from healthy individuals and stimulated them in vitro with anti-CD3 and anti-CD28 mAbs. CD39 expression was efficiently induced when stimulation was sustained during in vitro culture (chronic stimulation) (Figure 2E). In contrast, CD39 was expressed in smaller proportions of cells when stimulation was transient (24 h; acute stimulation) (Figure 2E). These results are in agreement with the activated and proliferative state of CD39^+^ cells (Figure 2A–C) and support their participation in an ongoing immune response involving chronic stimulation, potentially against tumor Ags.

### 3.3. The Circulating PD-1^+^CD39^+^ Memory CD4 T-Cell Subpopulation Contains Tumor-Ag-Specific Exhausted Cells and Predicts the Response to PD-1/PD-L1 Axis Blockade

The expression of ICs, PD-1 and TIGIT, and of CD39 as well as that of activation and proliferation markers in a population found at higher proportions in cancer patients than in HDs suggested that it could encompass exhausted tumor-Ag-specific T cells. We thus functionally characterized these cells in terms of exhaustion and Ag specificity.

**Figure 3:**
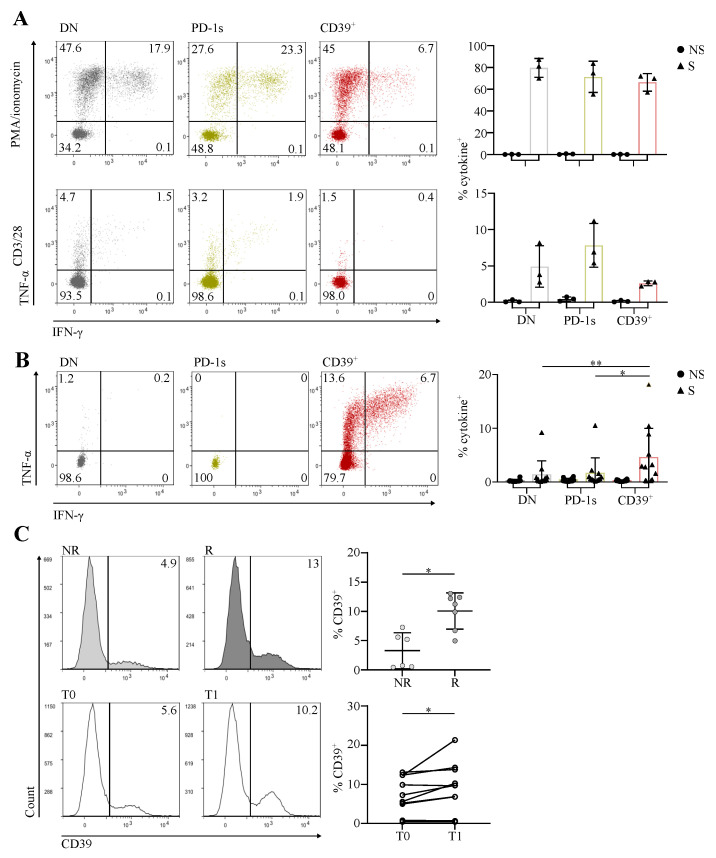
The circulating PD-1^+^CD39^+^ memory CD4 T-cell subpopulation contains tumor-Ag-specific exhausted cells and predicts the response to PD-1/PD-L1 axis blockade. (**A**) CD4 T cells isolated ex vivo from PBMCs of HNSCC patients (*n* = 3) were sorted by flow cytometry into the memory Tconv PD-1^-^CD39^-^ (DN), PD-1^+^CD39^-^ (PD-1s), and CD39^+^ populations. Cells were stimulated (S) with PMA/ionomycin (upper panels) or plate bound anti-CD3 and antiCD28 mAbs (lower panels), or not stimulated (NS), and cytokine production was assessed by intracellular staining. Representative dot plots of IFN-γ and TNF-α staining are shown for the three populations from one patient and the proportions of cytokine^+^ (IFN-γ^+^ and/or TNF-α^+^) cells are summarized for all cell populations and patients. (**B**) CD4 T cells isolated ex vivo from PBMCs of CC or HNSCC patients with human-papillomavirus (HPV)^+^ tumors (*n* = 9) were sorted by flow cytometry into the memory Tconv DN, PD-1s, and CD39^+^ populations. Sorted cells were stimulated in vitro with HPV E6 and E7 peptide pools and cultured for 20 days, cultures were restimulated (S) or not (NS) with the same peptide pools, and cytokine production was assessed by intracellular staining. Representative dot plots of IFN-γ and TNF-α staining of stimulated cultures are shown for the three populations from one patient, and the proportions of cytokine^+^ (IFN-γ^+^ and/or TNF-α^+^) cells are summarized for all cell populations and patients, with (S) or without (NS) peptide restimulation. (**C**) CD4 T cells, isolated ex vivo from pre- and posttreatment PBMCs of CC and anal cancer patients with HPV^+^ tumors receiving anti-PD-1/PD-L1, were stained and analyzed by flow cytometry. Upper panel, histograms show examples of CD39 expression in memory Tconv in immunotherapy non-responder (NR) and responder (R) patients in pretreatment PBMCs, and the proportions of CD39^+^ cells among pretreatment memory Tconv are summarized for all patients (*n* = 13). Lower panel, histograms show examples of CD39 expression in memory Tconv from pretreatment (T0) and posttreatment (T1) PBMCs from one patient, and the proportions of CD39^+^ cells among pre- and posttreatment memory Tconv are summarized for all patients (*n* = 10).

When stimulated with PMA/ionomycin, which bypass TCR, CD39^+^ cells produced amounts of IFN-γ and/or TNF-α comparable to those produced by DN and PD-1s memory Tconv (Figure 3A). When stimulated with anti-CD3/CD28, instead, CD39^+^ cells showed lower cytokine production in comparison with the two other populations (Figure 3A), suggesting that they were exhausted [23].

To assess the Ag specificity of memory CD4 Tconv according to PD-1 and CD39 expression, we selected patients with HPV 16- or 18-induced HNSCC or CC and sorted ex vivo DN, PD-1s, and CD39^+^ memory Tconv cells from their PBMCs. Sorted cells were stimulated with long overlapping peptides covering the full-length sequences of the E6 and E7 Ags of HPV 16 and/or 18, according to the HPV type identified in the autologous tumor. We assessed the expansion of specific T cells in day 20 cultures by intracellular cytokine staining following Ag restimulation. The highest proportions of cells producing IFN-γ and/or TNF-α in response to tumor Ag stimulation were found in cultures expanded from the CD39^+^ memory Tconv subpopulation (Figure 3B), indicating that it was enriched in patients in tumor-Ag-specific T cells.

We have shown that terminally exhausted CD8 T cells at the tumor site, which are tumor-Ag-specific, are predictive of the response to PD-1/PD-L1 axis blockade [4]. The expansion of early exhausted circulating CD8 T cells is also predictive of the response to anti-PD-1 in non-small-cell lung cancer [7]. Likewise, CD4 T cells expressing PD-1 and CD39 at the tumor site were shown by us and others to be exhausted and tumor-Ag-specific [8,9]. Based on these results, we hypothesized that the circulating exhausted, activated, proliferating, and tumor-Ag-specific CD39^+^ memory Tconv subpopulation we identified here could be predictive of the response to ICB. We thus assessed the proportions of CD39^+^ cells among circulating memory Tconv in patients receiving PD-1/PD-L1 blocking mAbs for the treatment of HPV-induced malignancies. The proportion of CD39^+^ memory Tconv at baseline was significantly higher in responder in comparison with non-responder patients (Figure 3C). In addition, the proportion of CD39^+^ memory Tconv was increased posttreatment in comparison with the baseline (Figure 3C).

## 4. Discussion

Identification of biomarkers of response to ICB relies on the understanding of the cellular and molecular mechanisms responsible for sensitivity or resistance of tumors to these therapies [1]. The adaptive T-cell response is the major target of PD-1/PD-L1 axis blockade and, understandably, most studies aiming to decipher resistance mechanisms and to identify biomarkers of response were focused on CD8 T cells, which directly recognize and kill tumor cells [24]. Whereas initial studies focused on the role of anti-PD-1/PD-L1 in reversing in situ terminal exhaustion of CD8 T cells, data obtained from preclinical tumor models and studies in cancer patients showed that responsiveness was dependent upon both reversal of exhaustion at the tumor site and proliferation of early exhausted peripheral CD8 T cells [4,24]. Expansion of peripheral CD8 T cells takes place in lymph nodes and is dependent upon CD4 help [16], inferring the importance of CD4 T cells in response to ICB. Interest in the CD4 response as a major player of immunotherapy efficacy was redoubled as the involvement of tumor-Ag-specific CD4 T cells at the tumor site in response to ICB began to be unveiled [15]. This was in agreement with prior studies showing the accumulation of tumor-Ag-specific T cells in tumors [16,25,26]. Based on these lines of evidence, we and others have investigated the exhaustion of CD4 T cells at the tumor site [8,9,13]. We showed that tumor-infiltrating PD-1^hi^CD39^+^ memory CD4 T cells are exhausted; tumor-Ag-specific; and, in response to anti-PD-1, enhance dendritic cells maturation, leading to tumor-Ag-specific CD8 T cells’ expansion [8]. Given the importance of the peripheral CD8 compartment in response to ICB [7], here, we investigated expression of ICs and of CD39 in circulating memory CD4 T cells. We show that PD-1^+^CD39^+^ memory CD4 T cells are enriched in the circulation of cancer patients in comparison with healthy individuals. These cells were activated and proliferating in vivo. In agreement with their IC expression profile, they showed functional exhaustion and, in patients with HPV-induced malignancies, they were enriched in tumor-Ag-specific cells. In patients receiving anti-PD-1/PD-L1 for the treatment of HPV-induced malignancies, circulating PD-1^+^CD39^+^ memory CD4 T cells were predictive of the response to therapy. Overall, our results suggest that companionship between exhausted tumor-Ag-specific CD4 and CD8 T cells could apply in the periphery as well as at the tumor site.

Terminally exhausted tumor-infiltrating CD8 T cells express tissue resident memory T-cell (Trm) markers, which allow for their retention at the tumor site [4,27,28]. In breast cancer patients, CD39-expressing exhausted CD8 T cells were detected in tumors, but not in the periphery [18]. We showed that tumor-Ag-specific CD8 T cells at the tumor site, which expressed the ICs PD-1, TIGIT, CTLA-4, and TIM-3, as well as CD39, are at an advanced effector memory (CD45RA^-^CCR7^-^) differentiation stage and, for the most part, lose CD28 expression [4]. In the periphery, in the same patients, CD8 T cells specific for the same Ags expressed PD-1 and TIGIT only were central memory (CD45RA^-^CCR7^+^) and expressed CD28 [4]. The circulating memory PD-1^+^CD39^+^ CD4 population that we identified here was composed of CD28^+^ cells. They expressed, for the most part, TIGIT, but did not express CTLA-4, which was expressed by PD-1^hi^CD39^+^ CD4 T cells at the tumor site [8]. These results argue in favor of the acquisition of PD-1 and CD39 expression by tumor-Ag-specific CD4 T cells in lymph nodes through chronic stimulation in the context of cancer, leading to the circulating PD-1^+^CD39^+^ memory CD4 T-cell population. We, however, cannot exclude that exhausted tumor-infiltrating CD4 T cells do recirculate. In support of that, we showed that, contrarily to terminally exhausted CD8 T cells, tumor-infiltrating exhausted CD4 T cells expressed only CD69, but no other Trm markers [8]. In humans, cutaneous resident memory CD4 T cells can downregulate CD69, exit tissue, and recirculate [29]. Of note, we detected very low expression of CD69 in circulating PD-1^+^CD39^+^ CD4 T cells. In addition, we showed that CD39^+^ memory Tconv TILs, like circulating CD39^+^ memory Tconv, contained high proportions of CD28^+^ cells. CD137 receptor, upregulated in T cells following activation [30], was associated, when expressed in CD8 and CD4 TILs, with clinical response to ICB [31]. In the circulation, CD137 expression in CD8, but not in CD4 T cells, was predictive of clinical responses [31,32]. Nonetheless, it would be of interest to assess the correlation between the expression of CD137 and of CD39 as well as their co-expression profile in circulating CD4 T cells and its possible association with patient responsiveness to ICB.

While the origin of circulating PD-1^+^CD39^+^ CD4 T cells remains to be determined, the fact that they reflect an immune response to ongoing disease, i.e., to tumors in cancer patients, as shown here, is in agreement with data obtained in individuals with chronic viral infections [33], where circulating virus-specific CD39^+^PD-1^+^ CD8 T cells are detected. These cells have an exhaustion signature and are detectable during HIV and HCV, but not CMV and EBV infection and, importantly, their proportions correlate with viral load [33].

## 5. Conclusions

We showed that PD-1 and CD39 co-expression in conventional memory CD4 T cells in blood could be used as a surrogate marker to unveil the existence of an active anti-tumor adaptive immune response. In cancer patients, PD-1^+^CD39^+^ memory CD4 T cells were tumor-Ag-specific, activated, and proliferating, yet exhibiting features of functional exhaustion. Their association with clinical responses to ICB suggests a potential central role of systemic CD4 T-cell help in facilitating tumor rejection under immunotherapy.

## Data Availability

Data supporting the reported results can be obtained from the corresponding author.

## Supplementary Materials

The following supporting information can be downloaded at https://www.mdpi.com/article/10.3390/cancers14153679/s1, Table S1: List of antibodies used for flow cytometry analyses and cell sorting; Table S2: List of overlapping peptides covering the full-length sequences of E6 and E7 proteins of HPV 16 and 18; Figure S1: Flow cytometry gating strategy for memory conventional CD4 T cells.
