# Supplementary material for: Circulating Exhausted PD-1+CD39+ Helper CD4 T Cells Are Tumor-Antigen-Specific and Predict Response to PD-1/PD-L1 Axis Blockade
Source: Cancers (Basel). 2022 Jul 28;14(15):3679. doi: 10.3390/cancers14153679 (PMC9367599; doi:10.3390/cancers14153679)
Supplement: Supplementary file 1 [file cancers-14-03679-s001.zip › cancers-1827208 - Supplementary.pdf]

cancers

MDPI

Supplementary Materials

Table S1. List of antibodies used for flow cytometry analyses and cell sorting.

Target	CloneCatalog	Number	Manufacturer
CD3	UCH-T1	563546	BD
CD3	UCH-T1	555333	BD
CD4	RPA-T4	560768	BD
CD197(CCR7)	150503	562555	BD
CD197(CCR7)	REA546	130-108-288	Miltenyi Biotec
CD45RA	HI100	11-0458-42	eBioscience
PD-1	EH12.1	565299	BD
PD-1	EH12.1	563789	BD
TIGIT	MBSA43	12-9500-42	eBioscience
CD366(TIM-3)	7D3	565562	BD
CD28	CD28.2	559770	BD
CD69	FN50	560737	BD
CD39	TU66	563681	BD
CD152(CTLA-4)	BNI3	563931	BD
FOXP3	PC5101	56-4776-41	eBioscience
CD25	B1.49.9	B09684	Beckman Coulter
CD127	HIL-7R-M21	560551	BD
INF-g	B27	557995	BD
TNF-a	cA2	130-096-613	Miltenyi Biotec
HLA-DR	LN3	47-9956-41	Thermo Fisher
KI67	B56	563576	BD
ICOS	C398.4A	313506	Biolegend

Table S2. List of overlapping peptides covering the full-length sequences of E6 and E7.proteins of HPV 16 and 18

HPV Type/Protein/Peptide	Sequence
HPV16 E6 1-15	MHQKRTAMFQDPQER
HPV16 E6 5-19	RTAMFQDPQERPRKL
HPV16 E6 9-23	FQDPQERPRKLPQLC
HPV16 E6 13-27	QERPRKLPQLCTELQ
HPV16 E6 17-31	RKLPQLCTELQTTIH
HPV16 E6 21-35	QLCTELQTTIHDIIL
HPV16 E6 25-39	ELQTTIHDIILECVY
HPV16 E6 29-43	TIHDIILECVYCKQQ
HPV16 E6 33-47	IILECVYCKQQLLRR
HPV16 E6 37-51	CVYCKQQLLRREVYD
HPV16 E6 41-55	KQQLLRREVYDFAFR
HPV16 E6 45-59	LRREVYDFAFRDLCI
HPV16 E6 49-63	VYDFAFRDLCIVYRD
HPV16 E6 53-67	AFRDLCIVYRDGNPY
HPV16 E6 57-71	LCIVYRDGNPYAVCD
HPV16 E6 61-75	YRDGNPYAVCDKCLK
HPV16 E6 65-79	NPYAVCDKCLKFYSK
HPV16 E6 69-83	VCDKCLKFYSKISEY
HPV16 E6 73-87	CLKFYSKISEYRHYC

HPV16 E6 77-91	YSKISEYRHYCYSLY
HPV16 E6 81-95	SEYRHYCYSLYGTTL
HPV16 E6 85-99	HYCYSLYGTTLEQQY
HPV16 E6 89-103	SLYGTTLEQQYNKPL
HPV16 E6 93-107	TTLEQQYNKPLCDLL
HPV16 E6 97-111	QQYNKPLCDLLIRCI
HPV16 E6 101-115	KPLCDLLIRCINCQK
HPV16 E6 105-119	DLLIRCINCQKPLCP
HPV16 E6 109-123	RCINCQKPLCPEEKQ
HPV16 E6 113-127	CQKPLCPEEKQRHLD
HPV16 E6 117-131	LCPEEKQRHLDKKQR
HPV16 E6 121-135	EKQRHLDKKQRFHNI
HPV16 E6 125-139	HLDKKQRFHNIRGRW
HPV16 E6 129-143	KQRFHNIRGRWTGRC
HPV16 E6 133-147	HNIRGRWTGRCMSCC
HPV16 E6 137-151	GRWTGRCMSCCRSSR
HPV16 E6 141-155	GRCMSCCRSSRTRRE
HPV16 E6 144-158	MSCCRSSRTRRETQL
HPV16 E7 1-15	MHGDTPTLHEYMLDL
HPV16 E7 5-19	TPTLHEYMLDLQPET
HPV16 E7 9-23	HEYMLDLQPETTDLY
HPV16 E7 13-27	LDLQPETTDLYCYEQ
HPV16 E7 17-31	PETTDLYCYEQLNDS
HPV16 E7 21-35	DLYCYEQLNDSSEEE
HPV16 E7 25-39	YEQLNDSSEEEDEID
HPV16 E7 29-43	NDSSEEEDEIDGPAG
HPV16 E7 33-47	EEEDEIDGPAGQAEP
HPV16 E7 37-51	EIDGPAGQAEPDRAH
HPV16 E7 41-55	PAGQAEPDRAHYNIV
HPV16 E7 45-59	AEPDRAHYNIVTFCC
HPV16 E7 49-63	RAHYNIVTFCCKCDS
HPV16 E7 53-67	NIVTFCCKCDSTLRL
HPV16 E7 57-71	FCCKCDSTLRLCVQS
HPV16 E7 61-75	CDSTLRLCVQSTHVD
HPV16 E7 65-79	LRLCVQSTHVDIRTL
HPV16 E7 69-83	VQSTHVDIRTLEDLL
HPV16 E7 73-87	HVDIRTLEDLLMGTL
HPV16 E7 77-91	RTLEDLLMGTLGIVC
HPV16 E7 81-95	DLLMGTLGIVCPICS
HPV16 E7 84-98	MGTLGIVCPICSQKP
HPV18 E6 1-15	MARFEDPTRRPYKLP
HPV18 E6 5-19	EDPTRRPYKLPDLCT
HPV18 E6 9-23	RRPYKLPDLCTELNT
HPV18 E6 13-27	KLPDLCTELNTSLQD
HPV18 E6 17-31	LCTELNTSLQDIEIT
HPV18 E6 21-35	LNTSLQDIEITCVYC
HPV18 E6 25-39	LQDIEITCVYCKTVL
HPV18 E6 29-43	EITCVYCKTVLELTE
HPV18 E6 33-47	VYCKTVLELTEVFEF
HPV18 E6 37-51	TVLELTEVFEFAFKD
HPV18 E6 41-55	LTEVFEFAFKDLFVV

HPV18 E6 45-59	FEFAFKDLFVVYRDS
HPV18 E6 49-63	FKDLFVVYRDSIPHA
HPV18 E6 53-67	FVVYRDSIPHAACHK
HPV18 E6 57-71	RDSIPHAACHKCIDF
HPV18 E6 61-75	PHAACHKCIDFYSRI
HPV18 E6 65-79	CHKCIDFYSRIRELR
HPV18 E6 69-83	IDFYSRIRELRHYSD
HPV18 E6 73-87	SRIRELRHYSDSVYG
HPV18 E6 77-91	ELRHYSDSVYGDTLE
HPV18 E6 81-95	YSDSVYGDTLEKLTN
HPV18 E6 85-99	VYGDTLEKLTNTGLY
HPV18 E6 89-103	TLEKLTNTGLYNLLI
HPV18 E6 93-107	LTNTGLYNLLIRCLR
HPV18 E6 97-111	GLYNLLIRCLRCQKP
HPV18 E6 101-115	LLIRCLRCQKPLNPA
HPV18 E6 105-119	CLRCQKPLNPAEKLR
HPV18 E6 109-123	QKPLNPAEKLRHLNE
HPV18 E6 113-127	NPAEKLRHLNEKRRF
HPV18 E6 117-131	KLRHLNEKRRFHNIA
HPV18 E6 121-135	LNEKRRFHNIAGHYR
HPV18 E6 125-139	RRFHNIAGHYRGQCH
HPV18 E6 129-143	NIAGHYRGQCHSCCN
HPV18 E6 133-147	HYRGQCHSCCNRARQ
HPV18 E6 137-151	QCHSCCNRARQERLQ
HPV18 E6 141-155	CCNRARQERLQRRRE
HPV18 E6 144-158	ARQERLQRRRETQV
HPV18 E7 1-15	MHGPKATLQDIVLHL
HPV18 E7 5-19	KATLQDIVLHLEPQN
HPV18 E7 9-23	QDIVLHLEPQNEIPV
HPV18 E7 13-27	LHLEPQNEIPVDLLC
HPV18 E7 17-31	PQNEIPVDLLCHEQL
HPV18 E7 21-35	IPVDLLCHEQLSDSE
HPV18 E7 25-39	LLCHEQLSDSEEEND
HPV18 E7 29-43	EQLSDSEEENDEIDG
HPV18 E7 33-47	DSEEENDEIDGVNHQ
HPV18 E7 37-51	ENDEIDGVNHQHLPA
HPV18 E7 41-55	IDGVNHQHLPARRAE
HPV18 E7 45-59	NHQHLPARRAEPQRH
HPV18 E7 49-63	LPARRAEPQRHTMLC
HPV18 E7 53-67	RAEPQRHTMLCMCCK
HPV18 E7 57-71	QRHTMLCMCCKCEAR
HPV18 E7 61-75	MLCMCCKCEARIKLV
HPV18 E7 65-79	CCKCEARIKLVVESS
HPV18 E7 69-83	EARIKLVVESSADDL
HPV18 E7 73-87	KLVVESSADDLRAFQ
HPV18 E7 77-91	ESSADDLRAFQQLFL
HPV18 E7 81-95	DDLRAFQQLFLNTLS
HPV18 E7 85-99	AFQQLFLNTLSFVCP
HPV18 E7 89-103	LFLNTLSFVCPWCAS
HPV18 E7 91-105	LNTLSFVCPWCASQQ

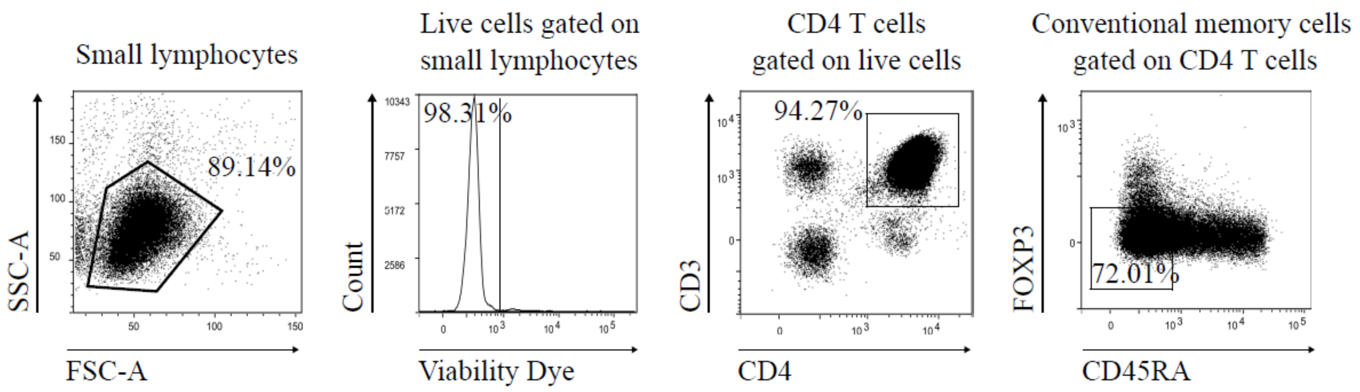

Figure S1. Flow cytometry gating strategy for memory conventional CD4 T cells. CD4 T cells. magnetically sorted *ex vivo* from PBMCs were stained with the viability dye (Fixable Viability Dye eFluor™ 506, eBioscience™) then with mAbs specific for CD3, CD4, CD45RA and FOXP3 and were analyzed by flow cytometry. The dot plots and histogram show the gating strategy used to identify memory ($CD45RA^-$) conventional (Tconv; $FOXP3^-$) CD4 T cells ($CD3^+CD4^+$).
